# Large Number Discrimination by Mosquitofish

**DOI:** 10.1371/journal.pone.0015232

**Published:** 2010-12-22

**Authors:** Christian Agrillo, Laura Piffer, Angelo Bisazza

**Affiliations:** Department of General Psychology, University of Padova, Padova, Italy; Université Pierre et Marie Curie, France

## Abstract

**Background:**

Recent studies have demonstrated that fish display rudimentary numerical abilities similar to those observed in mammals and birds. The mechanisms underlying the discrimination of small quantities (<4) were recently investigated while, to date, no study has examined the discrimination of large numerosities in fish.

**Methodology/Principal Findings:**

Subjects were trained to discriminate between two sets of small geometric figures using social reinforcement. In the first experiment mosquitofish were required to discriminate 4 from 8 objects with or without experimental control of the continuous variables that co-vary with number (area, space, density, total luminance). Results showed that fish can use the sole numerical information to compare quantities but that they preferentially use cumulative surface area as a proxy of the number when this information is available. A second experiment investigated the influence of the total number of elements to discriminate large quantities. Fish proved to be able to discriminate up to 100 vs. 200 objects, without showing any significant decrease in accuracy compared with the 4 vs. 8 discrimination. The third experiment investigated the influence of the ratio between the numerosities. Performance was found to decrease when decreasing the numerical distance. Fish were able to discriminate numbers when ratios were 1∶2 or 2∶3 but not when the ratio was 3∶4. The performance of a sample of undergraduate students, tested non-verbally using the same sets of stimuli, largely overlapped that of fish.

**Conclusions/Significance:**

Fish are able to use pure numerical information when discriminating between quantities larger than 4 units. As observed in human and non-human primates, the numerical system of fish appears to have virtually no upper limit while the numerical ratio has a clear effect on performance. These similarities further reinforce the view of a common origin of non-verbal numerical systems in all vertebrates.

## Introduction

During the last decade numerous studies have documented that numerical competence is not uniquely human. Field studies have documented that being able to process numerical information is advantageous in several ecological contexts [1], [2] while laboratory studies have provided evidence of rudimentary numerical abilities in animals as diverse as mammals [3], [4], [5], [6], birds [7], [8], [9], amphibians [10], fish [11], [12], [13] and social insects [14].

Comparative research suggests that mammals and birds share an approximate system of numerical representation [15], [16], [17]. This is proposed to be an analog magnitude system for approximate numerical estimation that obeys Weber's law, which maintains that, as numerical magnitude increases, a larger disparity is needed to obtain the same level of discrimination. In discriminating two sets of objects, the performance is therefore expected to be strongly dependent on the ratio of two numerosities and minimally affected by the total number of objects in the sets. For example, Lipton and Spelke [18] found that 6-month old infants can discriminate between 8 and 16 tones (1∶2 numerical ratio), while performance dropped to chance level when 8 and 12 tones were presented (2∶3 ratio). Hauser and colleagues [19] reported similar results in rhesus monkeys. Using familiarization–discrimination paradigm it has been observed that cotton-up tamarins can successfully discriminate between 4 and 6 syllables (2∶3 ratio) but not between 4 and 5, suggesting that the limit of large number discrimination in monkeys, as in human infants, is set by the numerical ratio.

Some evidence suggests that human adults, infants, and non-human primates may possess a second non-verbal numerical system that allows rapid and precise representation of a small number of objects [20], [21], [22]. This system has been proposed to depend on a mechanism for representing and tracking small numbers of individual objects [20], [21], [23]. Since it operates by keeping track of individual elements, it is precise but allows for the parallel representation of up to 3–4 elements [21], [22], [24]. Some studies have however questioned the hypothesis of a separate cognitive mechanism for representing small sets of objects. A study found that infants' discrimination of auditory events was affected by numerical ratio even for small values, suggesting that infants can use analog magnitudes for both small and large quantities in the auditory domain [25]. In a task requiring the ordering of pairs of numerosities, Cantlon and Brannon [15] found in adult humans and rhesus monkeys that accuracy and reaction time were ratio-dependent for both small and large quantities, in agreement with the existence of a single non-verbal mechanism over the whole numerical range.

From the above account it is evident that the issue whether primates possess a single or two specialized numerical systems is currently highly debated. Although there is even less evidence for the existence of separate quantificational systems in organisms other than primates (but see [26], [27]), at this stage of understanding, it is prudent even investigating lower vertebrates to study the discrimination of small and large numbers separately.

Numerical abilities have recently been investigated in fish by exploiting the natural tendency of social species to select the more numerous of two available social groups [13], [28], [29], [30]. A comprehensive study conducted on mosquitofish using this paradigm revealed that the numerical abilities of fish closely resemble those previously reported for primates [11]. In detail, fish discriminated between shoals differing by one unit when the paired numbers were 1 vs. 2, 2 vs. 3 and 3 vs. 4, while no capacity has been reported for larger numerical comparisons (i.e. 4 vs. 5 or 5 vs. 6). On the other hand, mosquitofish were also able to distinguish between shoals containing more than 4 individuals, provided that the numerosity ratio was at least 1∶2, such as 4 vs. 8.

Numerosity usually co-varies with several other attributes such as the cumulative surface area, the overall space occupied by the sets or the density of the elements. Humans and non-human animals can use the relative magnitude of these non-numerical cues to estimate which group is larger/smaller and the type of non-numerical cue used has been found to vary across species and context [4], [31], [32], [33], [34]. Taking into account these confounding factors represents one of the major challenges in the study of non-verbal numerical cognition.

Although, in a new study, mosquitofish were shown to be able to select the larger social group even after the access to non-numerical cues was made difficult [35], the confounding effect of continuous variables remains complicated to control in the social choice paradigm. Recently we have tackled this problem by examining discrimination of abstract geometric figures [12]. Mosquitofish were initially trained to discriminate between two small sets of figures (2 vs. 3) where no control for continuous variables was made. In the test phase continuous variables were controlled one at a time, to determine which cue the fish used during the learning process. Results showed that some continuous variables (such as total brightness of the stimuli and the sum of perimeters) were irrelevant, while performance dropped to chance level when stimuli were matched for cumulative surface area and the overall space occupied by the sets, suggesting that these two latter cues were spontaneously used by the fish during discrimination of small quantities. In a subsequent experiment subjects proved to be able to discriminate 2 from 3 objects after all non-numerical continuous variables were simultaneously controlled, providing the first direct evidence of use of numerical information in fish, at least in the small quantity range.

Currently no study has investigated whether fish can learn to discriminate contrasts other than 2 vs. 3 and in particular we have no information about the ability of fish to discriminate between large sets of objects. In the present study we used the same training procedure to study large number discrimination. In particular we aimed to assess whether 1) mosquitofish can be trained to discriminate quantities beyond the small quantity range, 2) they spontaneously rely on number or on continuous cues that co-vary with number and if these cues are the same used in the 2 vs. 3 discrimination, 3) there is an upper limit in the number of objects that can be processed by fish, and 4) as in mammals, performance is affected by the ratio between the numerosities to compare.

The purpose of the first experiment was to determine which cues mosquitofish used spontaneously when both numerical information and continuous variables are available (exp. 1.a). Subjects learned discrimination between 4 and 8 objects in the absence of any manipulation of the stimuli; after subjects reached learning criterion they were tested without reward while controlling one continuous variable at a time. In experiment 1.b, we trained fish to discriminate between 4 and 8 figures while continuous variables were simultaneously controlled, in order to determine whether fish could learn discrimination by using only numerical information. The second experiment was set up to investigate whether the number of objects to discriminate affects performance and if there is an upper limit in the number of elements that a mosquitofish can process. Fish were initially trained to discriminate between quantities with a 1∶2 numerical ratio and similar total numerosity (4 vs. 8, 5 vs. 10 and 6 vs. 12) and, in the test phase, they were presented with three numerical contrasts, again with a 1∶2 ratio, but differing in the total numerosity (4 vs. 8, 15 vs. 30 and 100 vs. 200 figures). In the third experiment we investigated the effect of the numerical ratio. Fish were trained to discriminate a 1∶2 ratio and, in the test phase, they were presented with three novel numerical contrasts that had similar total numerosity but different numerical ratios (1∶2, 2∶3 and 3∶4).

## Results

### Experiment 1a. Cues spontaneously used by fish to discriminate between large quantities

The procedure was identical to that adopted in a previous work investigating the mechanisms underlying small quantity discrimination [12]. We trained 10 female mosquitofish to discriminate between two numerosities. Each subject was inserted in an unfamiliar tank and trained to discriminate between two doors in order to rejoin its social group. Doors were associated with a pair of stimuli consisting of 4 or 8 geometric figures. These figures were randomly selected from a pool of approximately 100, and no control for non-numerical continuous variables was made in the learning phase. Six trials per day for a maximum of 10 days were provided. Once a subject had reached the learning criterion, it was admitted to the test phase in the same apparatus without reward (i.e. no possibility to rejoin the conspecifics) while controlling one continuous variable at a time. We controlled those variables that have been suggested as potentially relevant cues by previous studies on large quantity discrimination of vertebrates, namely the cumulative surface area, density, overall space occupied by the arrays and total brightness of the stimuli (see materials and methods).

**Figure 1:**
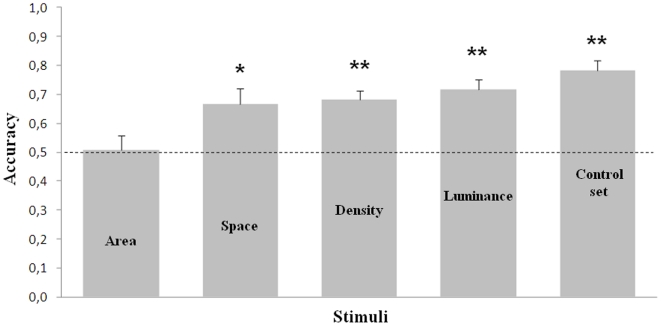
Results of Experiment 1a. Accuracy when luminance, density, overall space and cumulative surface area were controlled. Fish performance dropped to chance level only when cumulative surface area was paired (*  =  p < 0.05; **  =  p < 0.01).

All ten subjects reached the learning criterion in the training phase. We reported no difference in the accuracy (proportion of correct choices) between fish trained with 8 (mean ± std. dev.: 0.753±0.556) and those trained with 4 figures as positive (0.796±0.071; independent t-test t(8)  =  −1.06, p = 0.319). In the test phase significant discrimination was observed when no continuous variable was controlled (one sample t-test t(9)  = 7.97, p<0.001), when total brightness (t(9)  = 6.09, p<0.001), density (t(9)  = 6.13, p<0.001) and overall space occupied by the arrays (t(9)  = 3.0, p = 0.015) were singly controlled. On the contrary, no significant choice toward the trained quantity was found when cumulative surface area was controlled (t(9)  = 0.17, p = 0.872, Fig. 1) suggesting that this latter cue had been used by the subjects during the learning phase.

### Experiment 1b. Discrimination of large quantities by numerical information only

We trained 11 female mosquitofish to discriminate between 4 and 8 figures while we simultaneously controlled stimuli for continuous variables both in the pre-training and the training phase, with the aim of assessing whether fish could learn discrimination using numerical information only. Using the same geometric figures as the previous experiment, we designed pairs of stimuli in which cumulative surface area, density, overall space occupied by the arrays and total brightness were matched between the groups.

We found no difference in the accuracy between fish trained with 8 (0.671±0.089) and those trained with 4 figures as positive (0.683±0.025, t(10)  = 0.29, p = 0.777). Ten fish out of 11 reached the criterion (chi square test, p<0.05), proving thus able to select the trained numerosity. Overall the choice for the trained stimuli was significant (0.677±0.065, t(10)  = 9.04, p<0.001).

As a by-product of controlling continuous variables, stimuli differed for another non-numerical variable that the fish could have used instead of the number. In particular the by-product of pairing cumulative surface area was that smaller-than-average figures were more frequent in sets with 8 elements than in sets with 4. Therefore, after reaching the criterion, fish were subjected to a test phase without reinforcement using pairs of stimuli composed of figures of identical size. One subject was excluded from this test due to poor health and one did not reach the criterion in the training phase, hence 9 started the control test. Results showed that fish still significantly selected the trained numerosity, also when stimuli were made of identical geometric figures (0.616±0.112, t(8)  = 3.11, p = 0.015).

### Experiment 2. Influence of total number of elements

Subjects (n = 6) were initially trained to discriminate a 1∶2 numerical ratio by using 3 slightly different contrasts (4 vs. 8, 5 vs. 10 and 6 vs. 12). All stimuli were controlled for continuous variables. We found no difference in the accuracy between fish trained with the larger numerosity (0.685±0.032) and those trained with the smaller numerosity as positive (0.667±0.056, t(4)  = 0.50, p  = 0.643). Overall the choice for the trained stimuli is significant (0.676±0.041, t(5)  = 10.30, p<0.001).

**Figure 2:**
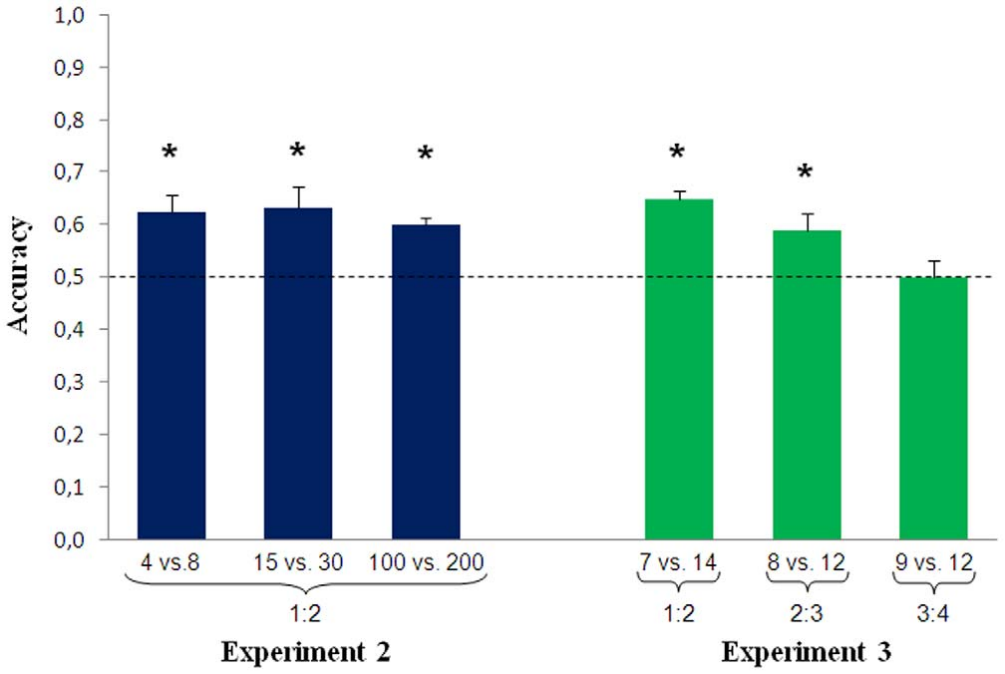
Results of Experiments 2 and 3. Fish performance is not affected by total numerosity when the numerical ratio is kept constant. On the contrary, numerical ratio does affect fish performance when total numerosity is controlled (*  =  p<0.05).

At day 4, subjects started the test phase. Fish were trained with 3 numerical contrasts that were identical in ratio (1∶2) but different in total numerosity (4 vs. 8, 15 vs. 30 and 100 vs. 200). We found no difference in the accuracy between fish trained with the larger numerosity (0.639±0.054) and those trained with the smaller numerosity as positive (0.600±0.033, t(4)  = 1.07, p = 0.346). A significant discrimination was found in all three conditions (4 vs. 8: t(5)  = 4.04, p = 0.010; 15 vs. 30: t(5)  = 3.32, p = 0.021; 100 vs. 200: t(5)  = 7.75, p = 0.001) with no significant difference among them (F(2,10)  = 3.35, p = 0.712, Fig. 2).

As in experiment 1b, we set up a control test without reinforcement presenting identical figures in a 100 vs. 200 object discrimination, to assess whether fish have learned discrimination by using a non-numerical strategy. Results proved that fish significantly distinguished between 100 and 200 also when stimuli were made of identical geometric figures (0.625±0.053, t(5) = 5.81, p = 0.002).

### Experiment 3. Influence of numerical ratio

Subjects (n = 6) were initially trained with two different numerical contrasts (5 vs. 10, and 6 vs. 12) having the same numerical ratio (1∶2). All the stimuli were controlled for continuous variables. We found no difference in the accuracy between fish trained with the larger numerosity (0.592±0.064) and those trained with the smaller numerosity as positive (0.648±0.085, t(4)  =  − 0.91, p = 0.417). Overall the choice for the trained stimuli was significant (0.620±0.073, t(5)  =  3.99, p = 0.010).

At day 4 fish started the test phase by presenting 3 novel numerical contrasts with ratios of 1∶2, 2∶3 and 3∶4 (7 vs. 14, 8 vs. 12 and 9 vs. 12 respectively) while total numerosity was made irrelevant (i.e. 20–21 figures in each pair). We found no difference in the accuracy between fish trained with the larger numerosity (0.595±0.036) and those trained with the smaller numerosity as positive (0.563±0.014, t(4)  = 1.44, p = 0.224). A significant difference was found among the three numerical ratios (F(2,10) = 5.75, p = 0.022). Discrimination was successful with the 1∶2 numerical ratio and 2∶3 (respectively t(5)  = 8.73, p<0.001 and t(5)  = 2.71, p = 0.042), but not in 3∶4 numerical ratio (t(5)  = 0.0, p = 1.0, Fig. 2).

### Experiment 4. Comparison with adult humans

For comparison, the same stimuli presented to fish in experiment 2 and 3 were presented to 25 undergraduates that were required to estimate the larger of 2 sequentially presented groups of geometric figures while being prevented from using verbal counting. In half of the presentations stimuli were controlled for continuous variables (the set of stimuli presented to fish), while in the remaining half continuous variables could also suggest the larger set.

**Figure 3:**
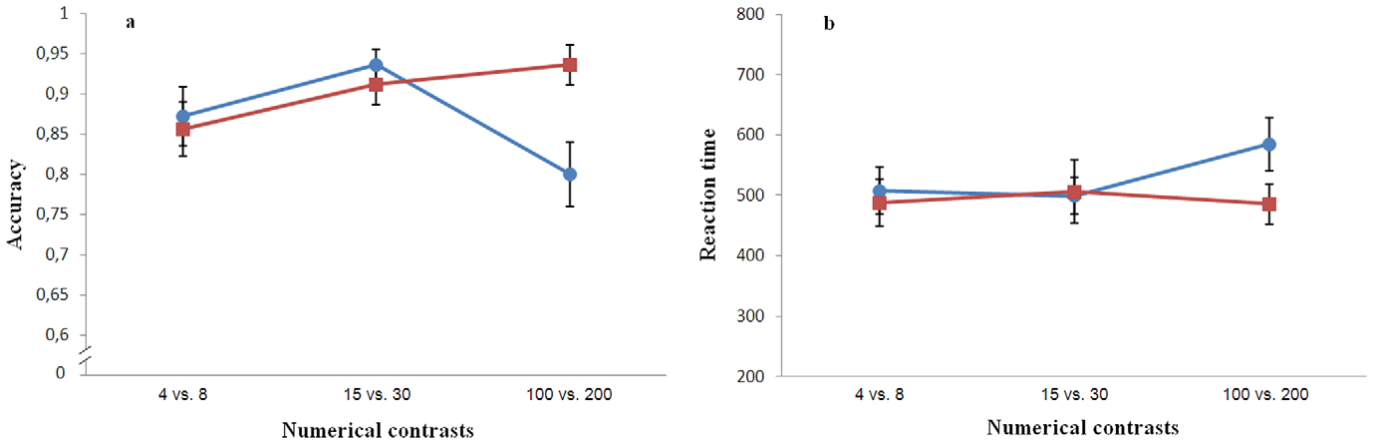
Results of Experiment 4. Adult humans were required to make numerical judgements with the same stimuli used in experiment 2. Both accuracy (graph a) and reaction time (ms, graph b) were not affected by total numerosity (Circles: stimuli controlled for continuous variables; squares: number and continuous variables available).

As regards the influence of the total number of elements to discriminate, accuracy and reaction time were analyzed separately by the 3×2 repeated measure ANOVA, with ‘Total numerosity’ (4 vs. 8, 15 vs. 30 and 100 vs. 200) and ‘Control of continuous variables’ (controlled/non-controlled) as within-subject factors. There was no effect of total numerosity or control of continuous variables on accuracy (Total numerosity: F(2,48)  = 2.29, p = 0.113; Control of continuous variables F(1,48)  = 2.09, p = 0.161). However, a significant interaction between the two factors was observed (F(2,48)  = 5.10, p = 0.010, Fig. 3.a), since accuracy slightly increased with increasing the number of elements in presentations not controlled for continuous variables, while it tended to decrease when participants were prevented from using continuous variables. When controlled and non-controlled presentations were analyzed separately, we found no significant effect of total numerosity in non-controlled presentations (repeated measure ANOVA F(2,48)  = 2.06, p = 0.139) while there was a significant decrease of accuracy with increasing total numerosity in controlled presentations (ANOVA F(2,48)  = 4.81, p = 0.012) due to a difference between the 15 vs. 30 and the 100 vs. 200 conditions (Bonferroni post hoc test, p = 0.014).

In reaction time (Fig. 3b) there was no significant main effect (Total numerosity F(2,48)  = 0.75, p = 0.479; Control of continuous variable F(1,48)  = 2.22, p = 0.149) or interaction (F(2,48)  = 1.60, p = 0.212).

**Figure 4:**
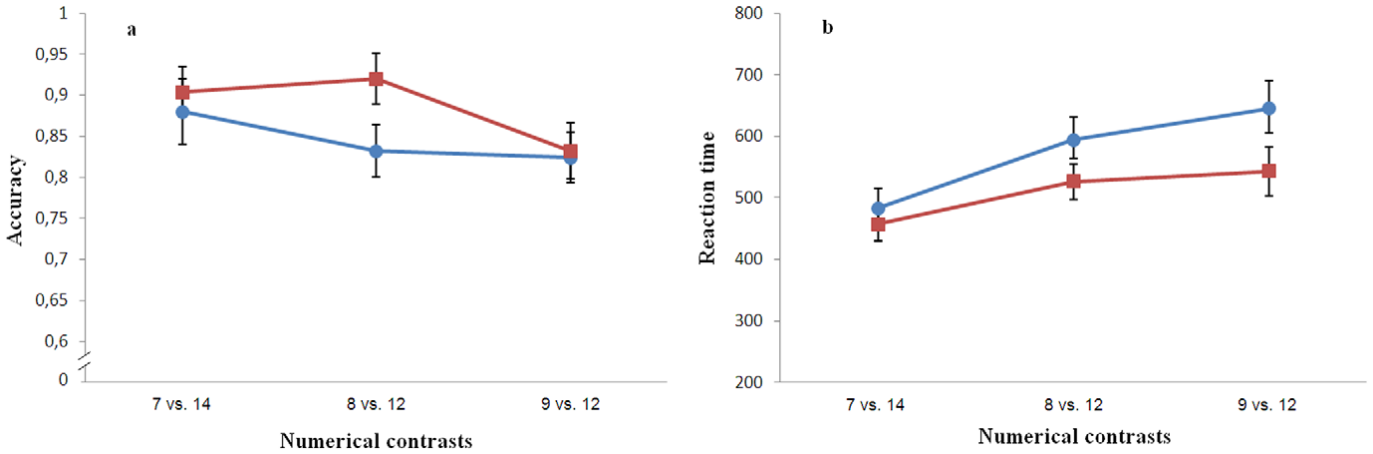
Results of Experiment 4. Adult humans were required to make numerical judgements with the same stimuli used in experiment 3. Both accuracy (graph a) and reaction time (ms, graph b) showed a significant sensitivity to numerical ratio (Circles: stimuli controlled for continuous variables; squares: number and continuous variables available).

Data on the influence of the numerical ratio were analyzed by 3×2 repeated measure ANOVA, with ‘Numerical ratio’ (1∶2, 2∶3 and 3∶4) and ‘Control of continuous variables’ as within-subject variables. A main effect of the numerical ratio on accuracy was observed (F(2,48)  = 4.37, p = 0.018) with the number errors increasing when the numerical distance between quantities decreased. The control of continuous variables and the interaction were not significant (respectively, F(1,48)  = 1.86, p = 0.185; F(2,48)  = 0.80, p = 0.455; Fig. 4a).

In reaction time there was a significant effect of both numerical ratio (F(2,48)  = 20.05, p<0.001) and control of continuous variable (F(1,48)  = 15.56, p<0.001, Fig. 4b). The latter was due to the fact that participants were slower to respond when the stimuli were controlled for continuous variables. The interaction was not significant (F(2,48)  = 1.53, p = 0.227).

## Discussion

Several recent studies have established that fish, like mammals and birds, display a rudimental capacity for estimating the numerical value of a set of objects. Few studies have however investigated the factors that affect such capacity and how fish compare to other vertebrates such as primates.

Previously we demonstrated that female mosquitofish can be trained to discriminate among small sets of abstract elements (2 vs. 3 elements). The results of the present study extend those findings showing that, like human adults, mosquitofish are able to compare different quantities of abstract elements beyond that small number range and can base their discrimination on numerical information only. Like humans, they improve their performance as the numerical distance between the numerosities increases, in accord with Weber's law, and are not affected by the variation in the total number of elements to discriminate, being able to select the larger of two sets even when they contained hundreds of elements.

### Number vs. continuous extent in large quantity discrimination

To assess which cue mosquitofish spontaneously use to discriminate two sets of elements, in experiment 1a subjects were first trained to a criterion with a set without a control for continuous variables and then tested in extinction while controlling one continuous variable at a time. We found that during the test phase the previous level of performance was maintained when the sets were matched for total brightness, density or overall space and, predictably, in the control set with no control of continuous variables. In contrast their performance dropped to chance level when stimuli were paired for cumulative surface area, thus indicating that this latter cue had been used during the learning process. Previous works have demonstrated that such a variable plays a key role in quantity discrimination both in humans [21], [36] and other mammal species [4], [32]. The same non-numerical cue was found to be important for mosquitofish when they had to select between two social groups differing in numerosity [11].

In a recent study we used the same procedure to assess the cues spontaneously used by mosquitofish when trained to discriminate between 2 and 3 figures [12] and we found that cumulative surface area was important also in the discrimination of small numbers. There is, however, an important difference with the results found here, specifically that the overall space occupied by the configuration was found to be an important cue in the 2 vs. 3 discrimination, while in the present study we found it to be irrelevant for the 4 vs. 8 discrimination. Differences in the cue used in the small number range and outside it were observed also in another study showing that mosquitofish spontaneously attended the overall quantity of movement within the shoal in the 4 vs. 8 fish discrimination, while they seemed to ignore this cue in a 2 vs. 3 fish comparison [11].

At present we can only speculate about the nature of these differences. One possibility is that, as suggested for primates, discrimination in the small number range (<4) and above it is based on different cognitive systems, a precise system for small numbers and an approximate system for large numbers [19], [21], [22]. However the difference between the two studies may be simply due to the fact that it is harder to uncorrelate numerical and non-numerical array parameters when dealing with small numbers, such as in the 2 vs. 3 comparison, than with larger numbers.

The fact that mosquitofish spontaneously use continuous variables does not necessarily imply that they are unable to discriminate two groups on the basis of numerosity alone. Indeed results of experiment 1b, in which the access to non-numerical cues was prevented, showed that fish can make discriminations beyond the small quantity range, such as 4 vs. 8, using the sole numerical information. The capacity to discriminate large numbers, previously reported for infants and non-human mammals, has been recently documented in birds. New Zealand robins for instance were proved to discriminate the same numerical contrast presented to fish (4 vs. 8) by using numerical information only [27]. Similarly, domestic chicks [8] are able to perform additions even beyond 4 units apparently without attending non-numerical cues. The list of vertebrate species able to enumerate large quantities is here extended to include a species, the eastern mosquitofish, which is phylogenetically very distant from mammals and possess a much smaller brain size.

### Influence of total number of elements

Experiment 2 was designed to study the influence of the total number of elements on discrimination performance. In the test phase of this experiment, fish trained to discriminate the larger (or smaller) of two numerosities learn to abstract this information and transfer it to novel numerosities, a cognitive ability considered one of the signatures of number representation in animal species [4], [37], [38]. Surprisingly in this experiment subjects could discriminate successfully all three numerical contrasts and showed no significant decrease in accuracy in the discrimination of 100 vs. 200 elements compared with the 4 vs. 8 object discrimination. A potentially confounding factor in this experiment is that, as a by-product of pairing the cumulative surface area, geometric figures larger than the average were more common in the set containing 100 elements than in the set containing 200 elements. Therefore, fish could have used a non-numerical strategy to learn this discrimination. However, the control trials carried out in extinction at the end of the training showed that fish also significantly selected the trained numerosity when stimuli were composed of identical elements, ruling out the possibility that they could have used the size of the single elements to learn the discrimination.

Previous studies have reported that trained monkeys can spontaneously extend a numerical rule learned with the values 1 through 9 to order pairs of the numerosities from 2 to 30 items [15] and rats could learn to select the correct box when its position is higher than 10^th^ in a row of 18 boxes [39]. As far as we know, results of experiment 2 represent the first evidence, in non-human species, of the capacity to compare very large numbers. The fact that fish can discriminate among hundreds of objects supports the notion that the mechanisms underlying large quantity discrimination have no upper limit and our results also suggest that discrimination of very large numerosities is not more difficult for a fish than a discrimination involving few elements.

Some studies have suggested that humans may be able to make numerical judgements in textures containing large numbers of dots (over 300 dots: [36], [40]). However, intra-specific comparison and, in particular, comparison of humans and non-human species is generally difficult, since different studies use different stimuli and procedures. For this reason we tested a sample of undergraduate students in the same numerical judgement as fish while preventing them from using verbal counting. Interestingly, the tests with students essentially replicated the results previously obtained with fish. There is one main difference between students and fish. In the condition of the controlled non-numerical variables (the more similar to the fish experiment), when passing from the 15 vs. 30 to the 100 vs. 200 condition, the task became slightly more difficult for humans but apparently not for fish which appeared equally good at discriminating 100 from 200 figures as they were at comparing smaller quantities. Another study, testing adults in the comparisons 10 vs. 20, 15 vs. 30, 20 vs. 40 and 25 vs. 50 in 2 sensory modalities found that reaction time was not influenced by total numerosity in the visual modality while it increased with increasing numerosity in the auditory modality or across the modalities [41]. Our data do not necessarily mean that fish and humans differ. The difference between the two species may derive from the fact that the human data were more precise and based on a large number of subjects and therefore the fish data may lack enough statistical power to evidence the subtle differences found in the undergraduates' experiment.

### Influence of numerical ratio

Experiment 3 clearly demonstrated that in mosquitofish the capacity to compare numbers is modulated by their ratio, as predicted for the analog magnitude system. This result agrees with a large body of experimental evidence indicating that the discrimination of large numbers in animals improves in precision with the numerical distance between the quantities to compare, according to Weber's law. Ratio dependency appears broadly consistent across a variety of paradigms (i.e. preferential looking time, training or spontaneous choice test), stimuli (dots, conspecifics or food) and species [15], [16], [19], [41], [42], [43], [44]. A ratio dependency has been suggested to occur also for fish when choosing between social groups of different numerosities, although no control for non-numerical variables could be done in these studies [11], [13]. Many studies have shown that large number discrimination is ratio-dependent in adult humans [45], [46], [47]. Yet large differences in the kind of stimuli and in the procedure adopted make a direct comparison difficult. When we compared the performance of undergraduate students and mosquitofish in the same discriminations, we found that, despite obvious quantitative differences in performance (with humans being more accurate in all comparisons), humans and fish produced similar patterns of accuracy documenting similar sensitivity to numerical ratio in the two species.

The influence of numerical ratio has been extensively investigated also in infants. The ability to discriminate 2 numbers in our species is already displayed by 6-month olds, that are able to discriminate a 1∶2 numerical ratio (i.e. 8 vs. 16) but not a 2∶3 ratio [18]. The precision of the system continues to increase over development: 10-month olds are able to discriminate a 2∶3 but not a 4∶5 ratio, preschool children a 3∶4 ratio, 6-year olds a 5∶6 ratio and adults a 7∶8 ratio [41], [48], [49]. In our study, fish proved able to discriminate 1∶2 and 2∶3 numerical ratios, while the performance dropped to chance level with a 3∶4 numerical ratio. Thus the resolution of the analog magnitude system in fish appears approximately comprised between that of a 10-month old infant and that of a preschool child.

It might appear surprising that the mosquitofish's ability to discriminate large numerosities is similar to that observed in humans in almost all respects. Two different lines of reasoning can help to explain this. In the first place, it is quite apparent that the cognitive abilities and the complexity of behaviour of teleost fish have previously been greatly underestimated and it is now well documented that, in this group, we can observe many of the cognitive functions that were once believed to be associated with evolution of a large brain in mammals and birds. Fish, for instance, can recognize up to 40 familiar individuals, cooperate to achieve a common goal, remember the outcome of past cooperative interactions and bias future social decisions accordingly, copy the behaviour of others and learn new foraging and anti-predator habits from an expert conspecific, show cultural traditions, exploit information from observations of mating or aggressive interactions of other individuals, and use tools (reviewed in [50], [51], [52]).

Secondly, analysis of neural circuitry in species with a simple nervous system, such as insects, showed that a very small number of neurons may be sufficient to support cognitive functions that are apparently complex [53], such as the ability of enumerating. This is also supported by a recent study using artificial neural networks [54] where it has been shown that less than 25 units can be enough for evolved agents to represent quantities with a performance similar to that observed in living organisms such as amphibians or fish.

To synthesize, the numerical system studied here shows the signature of an analog magnitude system of representation of numbers that conforms to Weber's law [55]. Given the strong correspondence observed in this study in the performance of fish and adult humans tested in comparable situations and, more in general, the similarities in the discrimination of large numerical quantities between adults, infants, several non-human primates, birds, amphibians and fish, we suggest – as other authors have done [15], [47], [56], [57] – that all vertebrates may share the same quantificational systems inherited from a common ancestor.

## Materials and Methods

### Ethics Statement

Experiments involving animals (exp. 1, 2 and 3) comply with all laws of the country (Italy) in which they were performed (D.M. 116192) and were approved by ‘*Ministero della Salute*’ (permit number: C7-2006). The experiment with adult humans (exp. 4) was approved by the ethical committee of the Department of General Psychology of University of Padova and was conducted according to the Declaration of Helsinki. Before testing, all participants gave their written consent.

### Experiment 1a. Cues spontaneously used by fish to discriminate between large quantities

#### Subjects

Ten female mosquitofish (*Gambusia holbrooki*) were used as subjects. Only females were used in this and the following experiments since we used social reinforcement and females are much more social than males in this species [58]. Fish were collected from Valle Averto, a system of fresh and brackish water ponds in the Venetian lagoon basin (Italy), returned to the laboratory and stocked in small mixed-sex groups (10–20 fish, approx. 1∶1 sex ratio) kept in 75-l glass aquaria with abundant vegetation (*Vesicularia dubyana* and *Ceratophyllum demersum*), lit by a 20W fluorescent lamp (16L∶8D) and with a water temperature that was maintained at 25±2° C. Subjects were used once; companion females, on the other hand, were used more than once.

#### Experimental protocol

**Figure 5:**
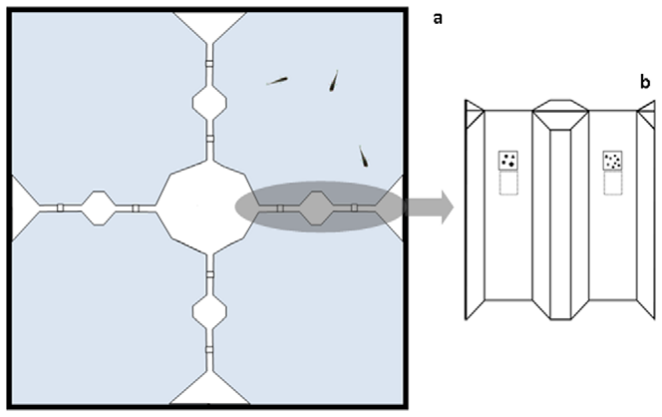
Schematic representation of the apparatus used in the pre-training phase. The tank was divided into four equal sectors by plastic partitions (a). Each partition contained two potential doors (b): only the door below the reinforced quantity permitted fish to pass from one sector to the other.

Since operant conditioning is normally a stressful procedure for fish, we adopted a pre-training procedure. Pre-training apparatus consisted of a 68×68×38 cm tank, divided into 4 equal sectors by white plastic partitions (Fig. 5). The tank was lit by 4 fluorescent lamps positioned around the perimeter, and water was maintained at a temperature of 25°±2°C. The bottom was covered with natural gravel and vegetation as well as aquarium filters.

To move between sectors, each partition contained 2 doors of equal size (2.5×3.5×1 cm) closed by a flexible transparent plastic material and located 12.5 cm from the floor of the tank, with a distance of 8 cm between them. Above each of the 2 doors we placed 2 stimuli, each occupying a 3×3 cm area. Each stimulus set contained one exemplar with 4 elements and one with 8. Elements were geometric figures differing in shape, size and luminance, randomly chosen from a set of approximately 100 elements and positioned on a white background.

Only the door below the reinforced quantity permitted them to pass from one sector to the other. This was achieved by gluing the flexible transparent material on the top of the door, so that fish could easily bend it and pass through the door. On the other door, the transparent material was glued also at the bottom, so that fish could not pass through. An unblocked door could be traversed in both directions, and pairs of stimuli were placed on both sides of the partition so that a total of 8 different pairs were presented inside the tank at the same time. These stimuli were changed daily, therefore a total of 56 different pairs of stimuli were used during the pre-training phase.

**Figure 6:**
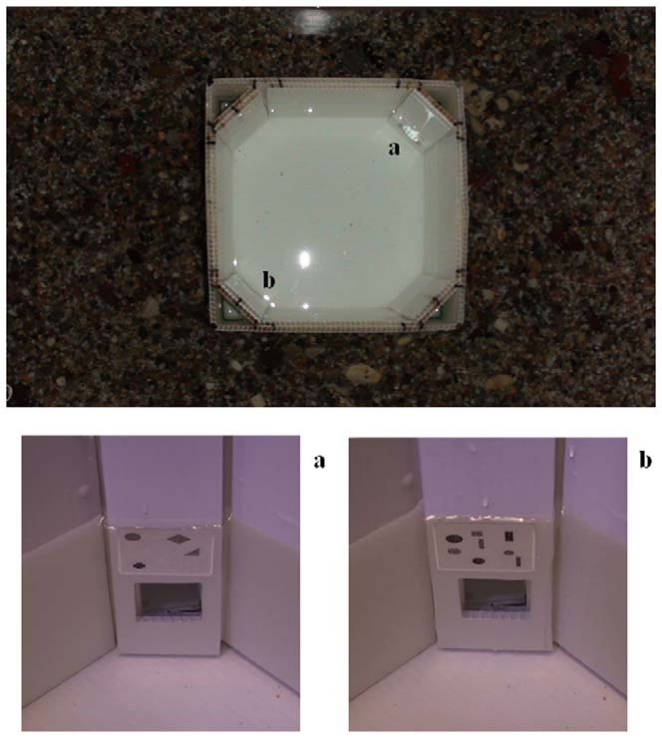
Apparatus used to train fish in experiment 1. Subjects were placed in the middle of a test chamber provided with two doors placed at opposite corners, one associated with 4 (a) and the other associated with 8 (b) figures. Only the door associated with the reinforced quantity could be opened by the fish in order to rejoin shoal mates in the outer tank.

The experimental apparatus was used in the training phase and in the following test phase (Fig. 6). It consisted of a small white test chamber (16×16×16 cm) inserted into a larger tank (60×26×36 cm) to provide a comfortable area with vegetation and food where the test fish were placed together with 3 other companion females 10 minutes before starting the training session. The tank was placed in a dark room and covered with a one-way screen to eliminate extra-tank cues. There is compelling evidence that female mosquitofish are highly social and spontaneously tend to join other females when placed in an uncomfortable environment [11], [58]. Previous work has shown that the herein described environment provides motivation for social reinstatement in mosquitofish [12].

At two corners of the chamber, two small tunnels (3×4×2.5 cm, located 2 cm from the floor of the tank) made from white plastic material were inserted, allowing the fish to pass through it to rejoin conspecifics in the outer tank. At the end of each tunnel there was a door similar to that used in the pre-training tank. As previously, one door was blocked, while the other could be opened by bending the flexible plastic material.

Sixty new pairs of stimuli were used, with the same characteristics of those used in the pre-training phase. As the elements of the stimuli were randomly selected, during both the pre-training and the training phase, fish could learn to distinguish between 2 quantities by using both number and non-numerical continuous variables that co-vary with number.

Conversely, in the control test, five different sets of stimuli were presented. We controlled one continuous variable at a time, namely the total brightness of the stimuli, the overall space occupied by the arrays (space encompassed by the figures), the density of the elements, and the cumulative surface area of the elements (summed area of figures). The fifth set was a control set of stimuli, in which no control for non-numerical variables was performed. All stimuli were created by using Microsoft Office 2007 and the area, space, density and brightness were controlled using TpsDig software.

Three different phases were planned: 1) pre-training, 2) training and 3) test. Half of the subjects were trained towards the larger quantity (8), whereas the second half were trained towards the smaller quantity (4). In the first phase, 3 subjects were kept for 7 days inside the pre-training tank and fish were simply left free to swim inside the 4 sectors without any interference from the experimenter for the whole period. At the beginning of day 8, all fish commenced the training phase in the experimental apparatus: subjects were singly tested each day, 6 trials per day from a minimum of 3 to a maximum of 10 days. During the trials, fish were brought to the test tank by inserting them into a transparent plastic cylinder (4.5 cm in diameter) and placing it in the centre of the test chamber. After 10 seconds, the cylinder was removed, leaving the fish in the middle of the test chamber. The first door they initially reached was recorded until the fish was able to exit and rejoin conspecifics (the maximum time allowed for exit was 20 minutes). Inter-trial intervals lasted 5 minutes, during which time the fish was allowed to shoal with the conspecifics; in the meantime the experimenter changed the pairs of stimuli. The location of the target quantity was exchanged at each successive trial. Furthermore, since the subject was disoriented between successive trials and no external cue was available, from the point of view of the fish the two corners were equivalent, reducing any possibility that the fish may have preferentially chosen one door by using the geometrical information of the environment.

The learning criterion was a statistically significant frequency of the correct choice estimated with the chi square test. Starting from day 3, we statistically analyzed the daily performance of the subject, and once discrimination reached significance it was admitted the next day to the following test phase. The procedure for the test phase was similar to that used during the training phase, with the exception that we adopted an extinction procedure [12], [32], [59] by keeping both doors blocked.

The first choice was recorded for a maximum period of 2 minutes. After this period, fish were released outside the test tank and could join their conspecifics; 5 minutes later, the subject was re-inserted into the test chamber in the presence of a new pair of stimuli. This phase lasted 6 days, with six trials per day, for a total of 36 trials overall. The 5 sets were randomly intermingled during each daily session. In addition, trials with the same stimuli used during training were intermixed with test trials in order to reinstate the motivation of the subjects. However, trials with the same stimuli used during training were discarded from data analyses. Statistical tests were conducted using SPSS 17.0.

### Experiment 1b. Discrimination of large quantities using only numerical information

#### Subjects and apparatus

A total of 11 female mosquitofish were tested (6 individuals were trained towards the larger numerosity and 5 trained towards the smaller). The apparatus was the same as for the previous experiment. Fish were reared in the same conditions described in experiment 1a.

#### Experimental protocol

The procedure for this experiment was similar to the previous one, with the exception that during the pre-training and training phases we used pairs of stimuli in which cumulative surface area, total brightness, density of elements and the overall space occupied by the arrays were simultaneously controlled. However, since density and overall space are inversely correlated, half of the set were controlled for the overall space occupied by the arrays, while the second half were controlled for the density of the elements.

The key phase for this experiment was the training phase, since we aimed to determine whether fish could learn discrimination after cumulative surface area, total brightness, density of elements and the overall space occupied of the arrays were controlled for. During the training phase of this experiment all subjects received the same number of trials, 36 (6 trials per day for a total of 6 days). As before, the criterion for discrimination was a statistically significant frequency of correct choices during the training phase.

However, by pairing the cumulative surface area we could have provided subjects with additional non-numerical cues that the fish could have used instead of numbers. In fact in the larger sets smaller-than-average figures were likely to be more frequent. Therefore, after reaching the criterion, we added a test phase in which we presented pairs of stimuli with an extinction procedure in which all elements were identical in size and shape (all circles, all stars, etc.). The fish received a total of 24 trials (6 trials per day, for 4 days).

### Experiment 2. Influence of total number of elements

#### Subjects and apparatus

A total of 6 female mosquitofish were tested (3 trained towards the larger numerosities, 3 towards the smaller). The apparatus was the same as for the previous experiment. Similarly, fish were reared in the same conditions described for experiment 1.

#### Experimental protocols

The procedure for this experiment was similar to the previous one. During pre-training fish were presented 3 different numerical contrasts (4 vs. 8, 5 vs. 10, and 6 vs. 12) with the same numerical ratio (1∶2). All the stimuli were controlled for continuous variables and numerical contrasts were randomly intermingled.

After pre-training, the fish started the training phase: the fish had to discriminate between the same numerical contrasts presented in the pre-training for 3 consecutive daily sessions. In the following test phase the fish were presented with 2 novel numerical contrasts (15 vs. 30 and 100 vs. 200). These novel contrasts were presented using larger stimuli (5×5 cm instead of 3×3). In addition, a control set (4 vs. 8) was also presented. All three numerical contrasts have the same ratio (1∶2) and were controlled for continuous variables. The test phase lasted 10 days (6 trials each day, for a total of 60 trials). Twenty trials were presented for each numerical contrast, 2 each day, randomly intermingled among the 3 sets of stimuli.

The key phase for this experiment was represented by the comparison of fish accuracy in the 3 numerical contrasts to see whether the capacity to discriminate between large sets was affected by total numerosity.

However, as experiment 1b, by pairing the cumulative surface area we could have provided subjects with additional non-numerical cues. Therefore, at the end of the test phase, we set up a control test without reinforcement presenting identical figures (all squares) in a 100 vs. 200 object discrimination, to assess whether fish have learned discrimination by using a non-numerical strategy. Fish received a total of 24 trials (6 trials per day, for 4 days).

### Experiment 3. Influence of numerical ratio

#### Subjects and apparatus

A total of 6 female mosquitofish were tested (3 trained towards the larger numerosities, 3 towards the smaller). The apparatus was the same as for the previous experiment. Similarly, fish were reared in the same conditions described above.

#### Experimental protocols

The procedure for this experiment was similar to the previous one. During pre-training the fish were presented with 2 different numerical contrasts (5 vs. 10 and 6 vs. 12) with the same numerical ratio (1∶2). All the stimuli were controlled for continuous variables and numerical contrasts were randomly intermingled. In the training phase the fish had to discriminate between the same numerical contrasts presented in the pre-training for 3 consecutive daily sessions.

In the following test phase the fish were presented with 3 novel contrasts (7 vs. 14, 8 vs. 12 and 9 vs. 12) differing in numerical ratios while the total numerosity of the sets was made irrelevant (a total figure of 20 or 21 presented within each pair). The test phase lasted 14 days (6 trials each day). Twenty-eight trials were presented for each numerical contrast, 2 daily trials for each set. The key phase for this experiment was represented by the comparison of fish accuracy in the 3 numerical ratios, in order to see whether fish accuracy was affected by numerical ratio.

### Experiment 4. Comparison with adult humans

#### Participants

A total of 25 undergraduate students between the ages of 21 and 26 (mean age 22.9) participated as volunteers. They were carried out at the Department of General Psychology, University of Padova. All participants had normal or corrected vision.

#### Stimuli and procedure

Each test comprised 40 pairs of stimuli. Black figures were presented differing in size; they appeared in the centre of the screen on a white background. Cumulative surface area, total brightness, density of elements and the overall space occupied were controlled for in half of the presentations (the same stimuli used in experiments 2 and 3 with the fish), while in half of the presentations were not controlled for. In test 1, three different numerical contrasts with different total numerosity were presented: 4 vs. 8, 15 vs. 30 and 100 vs. 200. In test 2, three different numerical contrasts differing in ratio were presented: 7 vs. 14, 8 vs. 12 and 9 vs. 12. Stimuli were displayed on a 17-inch monitor, using E-Prime software, in a darkened room.

After a period of adapting to the dark, a short familiarization training phase with feedback was presented. Participants initially read the experimental instructions on screen. A fixation cross then appeared in the centre of the screen for 1000 ms, then a group of figures was displayed in the centre of the screen for 150 ms. Following a 500 ms delay, participants saw another group for 150 ms. Participants had to estimate which one of the two groups was more numerous by pressing one of two keys on the keyboard. For half of the stimuli the larger group was presented first, for half of the stimuli the smaller group was presented first. They were instructed to make their responses as quickly and accurately as possible. Furthermore, to prevent verbal processing of the stimuli, verbal suppression was introduced during the test by asking participants to repeat continuously ‘abc’. No feedback was provided during the test. Both accuracy and reaction time were recorded.

## References

[1] McComb K, Packer C, Pusey A (1994). Roaring and numerical assessment in contests between groups of female lions, *Panthera leo*.. Animal Behaviour.

[2] Lyon BE (2003). Egg recognition and counting reduce costs of avian conspecific brood parasitism.. Nature.

[3] Beran MJ, Evans TA, Leighty KA, Harris EH, Rice D (2008). Summation and quantity judgments of sequentially presented sets by capuchin monkeys (*Cebus apella*).. American Journal of Primatology.

[4] Kilian A, Yaman S, von Fersen L, Güntürkün O (2003). A bottlenose dolphin discriminates visual stimuli differing in numerosity.. Learning & Behavior.

[5] Matsuzawa T (2009). Symbolic representation of number in chimpanzees.. Current Opinion in Neurobiology.

[6] West RE, Young RJ (2002). Do domestic dogs show any evidence of being able to count?. Animal Cognition.

[7] Brannon EM, Wusthoff CJ, Gallistel CR, Gibbon J (2001). Numerical subtraction in the pigeon: Evidence for a linear subjective number scale.. Psychological Science.

[8] Rugani R, Fontanari L, Simoni E, Regolin L, Vallortigara G (2009). Arithmetic in newborn chicks.. Proceedings of the Royal Society B-Biological Sciences.

[9] White DJ, Ho L, Freed-Brown G (2009). Counting chicks before they hatch: Female cowbirds can time readiness of a host nest for parasitism.. Psychological Science.

[10] Uller C, Jaeger R, Guidry G, Martin C (2003). Salamanders (*Plethodon cinereus*) go for more: Rudiments of number in an amphibian.. Animal Cognition.

[11] Agrillo C, Dadda M, Serena G, Bisazza A (2008). Do fish count? Spontaneous discrimination of quantity in female mosquitofish.. Animal Cognition.

[12] Agrillo C, Dadda M, Serena G, Bisazza A (2009). Use of number by fish.. PLoS ONE.

[13] Gomez-Laplaza LM, Gerlai R (2010). Can angelfish (*Pterophyllum scalare*) count?.

[14] Gross HJ, Pahl M, Si A, Zhu H, Tautz J (2009). Number-based visual generalisation in the honeybee.. PLoS ONE.

[15] Cantlon JF, Brannon EM (2006). Shared system for ordering small and large numbers in monkeys and humans.. Psychological Science.

[16] Ward C, Smuts BB (2007). Quantity-based judgments in the domestic dog (*Canis lupus familiaris*).. Animal Cognition.

[17] Al Ain S, Giret N, Grand M, Kreutzer M, Bovet D (2009). The discrimination of discrete and continuous amounts in African grey parrots (*Psittacus erithacus*).. Animal Cognition.

[18] Lipton JS, Spelke ES (2003). Origins of number sense: Large-number discrimination in human infants.. Psychological Science.

[19] Hauser MD, Tsao F, Garcia P, Spelke ES (2003). Evolutionary foundations of number: Spontaneous representation of numerical magnitudes by cotton-top tamarins.. Proceedings of the Royal Society of London Series B-Biological Sciences.

[20] Trick LM, Pylyshyn ZW (1994). Why are small and large numbers enumerated differently? A limited-capacity preattentive stage in vision.. Psychological Review.

[21] Feigenson L, Carey S, Hauser MD (2002). The representations underlying infants' choice of more: Object files versus analog magnitudes.. Psychological Science.

[22] Hauser MD, Carey S, Hauser LB (2000). Spontaneous number representation in semi-free-ranging rhesus monkeys.. Proceedings of the Royal Society B-Biological Sciences.

[23] Uller C, Carey S, Huntley-Fenner G, Klatt L (1999). What representations might underlie infant numerical knowledge?. Cognitive Development.

[24] Pylyshyn ZW, Storm RW (1988). Tracking multiple independent targets: Evidence for a parallel tracking mechanism.. Spatial Vision.

[25] vanMarle K, Wynn K (2009). Infants' auditory enumeration: Evidence for analog magnitudes in the small number range.. Cognition.

[26] Bonanni R, Natoli E, Cafazzo S, Valsecchi P (2010). Free-ranging dogs assess the quantity of opponents in intergroup conflicts.. http://dx.doi.org/10.1007/s10071-010-0348-3.

[27] Hunt S, Low J, Burns KC (2008). Adaptive numerical competency in a food-hoarding songbird.. Proceedings of the Royal Society B-Biological Sciences.

[28] Agrillo C, Dadda M (2007). Discrimination of the larger shoal in the poeciliid fish *Girardinus faicatus*.. Ethology Ecology & Evolution.

[29] Agrillo C, Dadda M, Bisazza A (2007). Quantity discrimination in female mosquitofish.. Animal Cognition.

[30] Buckingham JN, Wong BBM, Rosenthal GG (2007). Shoaling decisions in female swordtails: How do fish gauge group size?. Behaviour.

[31] Stevens JR, Wood JN, Hauser MD (2007). When quantity trumps number: Discrimination experiments in cotton-top tamarins (*Saguinus oedipus*) and common marmosets (*Callithrix jacchus*).. Animal Cognition.

[32] Pisa PE, Agrillo C (2009). Quantity discrimination in felines: A preliminary investigation of the domestic cat (*Felis silvestris catus*).. Journal of Ethology.

[33] Clearfield MW, Mix KS (1999). Number versus contour length in infants' discrimination of small visual sets.. Psychological Science.

[34] Emmerton J, Renner JC (2009). Local rather than global processing of visual arrays in numerosity discrimination by pigeons (*Columba livia*).. Animal Cognition.

[35] Dadda M, Piffer L, Agrillo C, Bisazza A (2009). Spontaneous number representation in mosquitofish.. Cognition.

[36] Durgin FH (1995). Texture density adaptation and the perceived numerosity and distribution of texture.. Journal of Experimental Psychology-Human Perception and Performance.

[37] Davis H, Perusse R (1988). Numerical competence in animals: Definitional issues, current evidence and a new research agenda.. Behavioral and Brain Sciences.

[38] Koehler O (1950). The ability of birds to “count”.. Bulletin of Animal Behavior.

[39] Suzuki K, Kobayashi T (2000). Numerical competence in rats (*Rattus norvegicus*): Davis and Bradford (1986) extended.. Journal of Comparative Psychology.

[40] Hollingsworth WH, Simmons JP, Coates TR, Cross HA (1991). Perceived numerosity as a function of array number, speed of array development, and density of array items.. Bulletin of the Psychonomic Society.

[41] Barth H, Kanwisher N, Spelke ES (2003). The construction of large number representations in adults.. Cognition.

[42] Flombaum JI, Junge JA, Hauser MD (2005). Rhesus monkeys (*Macaca mulatta*) spontaneously compute addition operations over large numbers.. Cognition.

[43] Tomonaga M, Matsuzawa T (2002). Enumeration of briefly presented items by the chimpanzee (*Pan troglodytes*) and humans (*Homo sapiens*).. Animal Learning & Behavior.

[44] Izard V, Sann C, Spelke ES, Streri A (2009). Newborn infants perceive abstract numbers.. Proceedings of the National Academy of Sciences of the United States of America.

[45] Halberda J, Mazzocco MMM, Feigenson L (2008). Individual differences in non-verbal number acuity correlate with maths achievement.. Nature.

[46] Revkin SK, Piazza M, Izard V, Cohen L, Dehaene S (2008). Does subitizing reflect numerical estimation?. Psychological Science.

[47] Cantlon JF, Brannon EM (2007). Basic math in monkeys and college students.. Plos Biology.

[48] Xu F, Arriaga RI (2007). Number discrimination in 10-month-old infants.. British Journal of Developmental Psychology.

[49] Halberda J, Feigenson L (2008). Developmental change in the acuity of the “Number sense”: The approximate number system in 3-, 4-, 5-, and 6-year-olds and adults.. Developmental Psychology.

[50] Bshary R, Wickler W, Fricke H (2002). Fish cognition: a primate's eye view.. Animal Cognition.

[51] Brown C, Laland KN (2003). Social learning in fishes: A review.. Fish and Fisheries.

[52] Bisazza A, Evans J, Pilastro A, Schlupp I (2010). Cognition.. Ecology and evolution of poeciliid fishes.

[53] Chittka L, Niven J (2009). Are bigger brains better?. Current Biology.

[54] Hope T, Stoianov I, Zorzi M (2010). Through neural stimulation to behavior manipulation: A novel method for analyzing dynamical cognitive models.. Cognitive Science.

[55] Feigenson L, Dehaene S, Spelke ES (2004). Core systems of number.. Trends in Cognitive Sciences.

[56] Jordan KE, Brannon EM (2006). A common representational system governed by Weber's law: Nonverbal numerical similarity judgments in 6-year-olds and rhesus macaques.. Journal of Experimental Child Psychology.

[57] Beran MJ (2008). The evolutionary and developmental foundations of mathematics.. Plos Biology.

[58] Bisazza A, Marin G (1995). Sexual selection and sexual size dimorphism in the Eastern mosquitofish *Gambusia holbrooki* (Pisces Poeciliidae).. Ethology Ecology & Evolution.

[59] Chiandetti C, Vallortigara G (2008). Is there an innate geometric module? Effects of experience with angular geometric cues on spatial re-orientation based on the shape of the environment.. Animal Cognition.

